# Comparing Prescriptive and Descriptive Gender Stereotypes About Children, Adults, and the Elderly

**DOI:** 10.3389/fpsyg.2018.01086

**Published:** 2018-06-26

**Authors:** Anne M. Koenig

**Affiliations:** Department of Psychological Sciences, University of San Diego, San Diego, CA, United States

**Keywords:** gender, stereotypes, prescriptions, children, adults, elderly, age

## Abstract

Gender stereotypes have descriptive components, or beliefs about how males and females typically act, as well as prescriptive components, or beliefs about how males and females should act. For example, women are supposed to be nurturing and avoid dominance, and men are supposed to be agentic and avoid weakness. However, it is not clear whether people hold prescriptive gender stereotypes about children of different age groups. In addition, research has not addressed prescriptive gender stereotypes for the elderly. The current research measured prescriptive gender stereotypes for children, adults, and elderly men and women in 3 studies to (a) compare how prescriptive gender stereotypes change across age groups and (b) address whether stereotypes of males are more restrictive than stereotypes of females. Students (Studies 1 and 2) and community members (Study 3), which were all U.S. and majority White samples, rated how desirable it was for different target groups to possess a list of characteristics from 1 *(very undesirable*) to 9 (*very desirable*). The target age groups included toddlers, elementary-aged, adolescent, young adult, adult, and elderly males and females. The list of 21 characteristics was created to encompass traits and behaviors relevant across a wide age range. In a meta-analysis across studies, prescriptive stereotypes were defined as characteristics displaying a sex difference of *d* > 0.40 and an average rating as desirable for positive prescriptive stereotypes (PPS) or undesirable for negative proscriptive stereotypes (NPS) for male or females of each age group. Results replicated previous research on prescriptive stereotypes for adults: Women should be communal and avoid being dominant. Men should be agentic, independent, masculine in appearance, and interested in science and technology, but avoid being weak, emotional, shy, and feminine in appearance. Stereotypes of boys and girls from elementary-aged to young adults still included these components, but stereotypes of toddlers involved mainly physical appearance and play behaviors. Prescriptive stereotypes of elderly men and women were weaker. Overall, boys and men had more restrictive prescriptive stereotypes than girls and women in terms of strength and number. These findings demonstrate the applicability of prescriptive stereotypes to different age groups.

## Introduction

Gender stereotypes are both descriptive and prescriptive in nature. That is gender stereotypes have *descriptive* components, which are beliefs about what men and women typically do. They also contain strong *prescriptive* components, or beliefs about what men and women should do (Fiske and Stevens, 1993; Cialdini and Trost, 1998). This prescriptive nature is assumed to stem from the high level of contact and interdependence between men and women (e.g., Fiske and Stevens, 1993), which not only allows perceivers to create estimates of how men and women actually act but also creates expectations for how they should act.

Prescriptive stereotypes can have positive and negative components: (a) positive prescriptive stereotypes (PPS) designate desirable behaviors that one sex is encouraged to display more than the other and (b) negative proscriptive stereotypes (NPS) designate undesirable behaviors that one sex should avoid more than the other. These proscriptive stereotypes often involve characteristics that are undesirable in either sex, but are permitted in one sex, while being proscribed for the other. For example, according to past research (Prentice and Carranza, 2002; Rudman et al., 2012b), women are supposed to be communal (warm, sensitive, cooperative; PPS for women) and avoid dominance (e.g., aggressive, intimidating, arrogant; NPS for women), and men are supposed to be agentic (assertive, competitive, independent; PPS for men) and avoid weakness (e.g., weak, insecure, emotional; NPS for men). Yet dominance and weakness, which are undesirable, negative traits, are tolerated in men or women, respectively.

The current research measures both prescriptive and descriptive gender stereotypes to answer several questions about their content and magnitude. One first basic question is whether gender stereotypes have prescriptive components not only for adult men and women, but for males and females across different age groups, from toddlers to the elderly. Assuming prescriptive stereotypes exist across these age groups, the current research addresses how both the content and magnitude of prescriptive gender stereotypes changes across age groups. In addition, the current research compares the magnitude of PPS and NPS for males and females within each age group.

### Adult prescriptive stereotypes

The fact that gender stereotypes are prescriptive is important to our perceptions of men and women because prescriptive stereotypes indicate approved (or disapproved) behavior. Violations of these prescriptions create strong reactions in perceivers. Whereas violations of descriptive stereotypes often cause surprise, given the person is not acting how the perceiver thought most men or women act, violations of prescriptive stereotypes create reactions of anger and moral outrage, because the person is not acting as they are supposed to act (Rudman and Glick, 2010).

Thus, descriptive gender stereotypes can lead to prejudice and discrimination based on a perceived incongruency between gender stereotypes and role requirements, and prescriptive stereotypes can also produce prejudice if individuals violate gender norms (e.g., Burgess and Borgida, 1999; Heilman, 2001; Eagly and Karau, 2002). Specifically, the angry, moral outrage created by the violation of prescriptive stereotypes can lead to backlash, or social or economic penalties for the stereotype violator (e.g., dislike or not being hired for a position). Rudman et al. (2012a,b) posit that backlash against both female and male targets works to maintain the status hierarchy and keep men in high status positions, but limits agentic women's access to these same positions. For example, women who violate prescriptive stereotypes by acting dominant are disliked and therefore less likely to be hired even though they are seen as competent (Rudman et al., 2012a). Men can also be the recipients of backlash when they violate prescriptive stereotypes by lacking agency and showing weakness (Moss-Racusin et al., 2010; see summary by Rudman et al., 2012a).

Because of this backlash effect, prescriptive stereotypes can predict prejudice, even when descriptive stereotypes do not. For example, when male and female targets had equivalent resumes participants' descriptive stereotypes did not predict evaluations of the targets, but prescriptive stereotypes did predict prejudice toward women pursuing masculine roles (Gill, 2004). Prescriptive stereotypes also create pressures on women and men to act in certain ways, and thus men and women avoid violating stereotypes or hide their non-conforming behavior to avoid penalties, which increases the rate of stereotypical behavior and perpetuates perceivers' stereotypes (Prentice and Carranza, 2004; Rudman and Glick, 2010; Rudman et al., 2012a). Thus, prescriptive stereotypes have important ramifications for behavior.

Whether these prescriptive stereotypes are more restrictive for adult men or women is unclear. Much research has investigated backlash toward women, perhaps because women are often held back from high status positions, which is seen as an important discriminatory outcome in society. However, there are several forms of evidence that suggest men's behaviors may be more restricted than women's in adulthood. For example, although they did not have a direct measure of prescriptive stereotypes, Hort et al. (1990) demonstrated that men were described in more stereotypical terms than women. Other evidence for a restrictive male stereotype stems from looking at the outcomes of stereotype violation. According to the status incongruity hypothesis, there are two prescriptive stereotypes that could create backlash for men (lacking agency and displaying weakness) and only one for women (displaying dominance; Rudman et al., 2012a). This argument suggests that men are viewed more negatively than women for violating gender norms because men loose status (while women gain status) with the violation (Feinman, 1984; Sirin et al., 2004), and status is seen as a positive, desirable outcome. In addition, theories about precarious manhood also suggest that men have to publically and repeatedly prove their strength to be called men because manhood is an uncertain, tenuous social status (Vandello and Bosson, 2013). Even a single feminine or unmanly act could discount a man's status as a man, resulting in avoidance of feminine behaviors. According to this logic, these pressures may create strong prescriptive stereotypes for men to act agentically and avoid weakness to be considered a man—a pressure that is not as strong for women. Lastly, a sexual orientation perspective also indicates that men would be judged more harshly for feminine behavior than women are for masculine behavior because (a) men who display feminine behaviors are more likely to be perceived as gay than women who display masculine behavior (e.g., Deaux and Lewis, 1984; Herek, 1984; McCreary, 1994; Sirin et al., 2004), and (b) gay men are perceived more negatively than lesbians (e.g., Kite and Whitley, 1996). Given all of these ideas, prescriptive stereotypes may be stronger for men as a way to avoid these negative outcomes of a loss of status, manhood, and perceptions of homosexuality. The current research quantifies prescriptive stereotypes for males and females to assess their content and magnitude and attempts to make comparisons across the stereotypes for males and females.

### Prescriptive stereotypes about children

Penalties for stereotype violations also occur for children who act in counterstereotypical ways. Several studies show that reactions from both child (e.g., Smetana, 1986; Levy et al., 1995) and adult (e.g., Feinman, 1981; Martin, 1990; Sandnabba and Ahlberg, 1999) respondents demonstrate more negative consequences (e.g., approval, evaluations) of counterstereotypical behavior from boys than girls ranging from ages 3 to 8 years old. This negative reaction toward boys is often stronger in men than women (e.g., Martin, 1990). Parents give little latitude for boys' behaviors but encourage both feminine behavior as well as masculine occupations and interests for girls, even complaining that their daughters can be “too girly” with pink, princess paraphilia (Kane, 2012). Boys who are “sissies” are especially negatively perceived, whereas girls who are “tomboys” have both feminine and masculine interests and traits and therefore do not violate gender stereotypes as strongly (Martin, 1990, 1995; Martin and Dinella, 2012). Boys also elicit negative reactions for shy behavior, presumably because this behavior violates the male gender role (Doey et al., 2014). As with adults, boys' behavior may be more restricted because of links between feminine behavior and homosexuality (e.g., Sandnabba and Ahlberg, 1999; Sirin et al., 2004). Thus, the consequences for violating stereotypes appear to be especially harsh for boys, and boys tend to be bounded by stricter rules of gender conformity and are subject to stronger “gender policing” than girls. These penalties, similar to backlash in the adult literature, suggest that violations of prescriptive stereotypes are at play. However, the research on children's norm violations does not frame the negative outcomes for counterstereotypical behavior in terms of violations of prescriptive stereotypes. In fact, it is not clear whether people even hold strong prescriptive gender stereotypes about children.

In one study that did address prescriptive stereotypes in children, Martin (1995) measured both descriptive and prescriptive gender stereotypes by asking adults how typical (measuring descriptive stereotypes) and how desirable (measuring prescriptive stereotypes) a list of 25 traits were for 4–7 year old boys or girls. As Martin (1995) predicted, the typicality ratings differed more often than the desirability ratings: The descriptive stereotypes indicated that boys and girls differed on 24 of 25 of the traits, which were selected to contain some masculine, feminine, and neutral items. Yet only 16 of the 25 traits showed sex differences in desirability: Martin (1995) found that boys should enjoy mechanical objects, be dominant, be independent, be competitive, like rough play, and be aggressive but avoid crying/getting upset or being frustrated (compared to girls). Girls should be gentle, neat/clean, sympathetic, eager to soothe hurt feelings, well-mannered, helpful around the house, and soft-spoken and avoid being noisy. Although there were fewer prescriptive than descriptive stereotypes about children in this research, these findings also show that prescriptive gender stereotypes exist for children of elementary-school age in ways that are consistent with adult prescriptive stereotypes.

Although prescriptive stereotypes may exist for younger ages, one could argue that younger people may not be held to as high of a standard for their behavior because they are considered to be more malleable than older targets (see Neel and Lassetter, 2015). To the extent that children are seen as still learning their gender roles and associated appropriate behaviors, people may be more lenient and prescriptive stereotypes might be weaker. On the other hand, adults' descriptive gender stereotypes of children were stronger than their descriptive stereotypes of adults (Powlishta, 2000), and the same effect may apply to prescriptive stereotypes resulting in stronger stereotypes of children. Thus, the magnitude of prescriptive gender stereotypes for children of different ages and how they compare to adult prescriptive gender stereotypes is unclear.

### Prescriptive stereotypes about other age groups

Once males and females are old enough to understand their gender roles, perceivers may be less lax about what is desirable behavior. Not only may older teens be seen as more in charge of their own behavior, but adolescence and young adulthood highlights differences between males and females in ways that were not relevant to children given the advent of puberty and the initiation of dating scripts. Thus, stereotypical self-perceptions and peer pressure for conformity to gender roles may intensify during adolescence for both males and females (Massad, 1981; Hill and Lynch, 1983; Galambos et al., 1990). This “gender intensification hypothesis” states that there is an acceleration of gender-differential socialization and increased pressure to conform during adolescence. However, it is unclear if these self-beliefs would transfer to adults' stereotypes of male and female teens. Based on these ideas, one could predict that prescriptive stereotypes adults hold are stronger for adolescents. Whether males' behaviors would still be more restricted is unclear. Some researchers argue that gender role pressures intensify at this age mostly for boys (Massad, 1981; Galambos et al., 1990), which is in line with ideas about precarious manhood, where boys have to continue to strive to become men through their public behavior whereas girls become women through the natural process of menstruation and other biological changes that occur in adolescence (Vandello and Bosson, 2013). However, other researchers suggest a confluence of factors increase pressures on girls' behavior in adolescence compared to childhood, with the leniency given to girls to be tomboys replaced with stricter gender norms and a pressure to exhibit feminine behaviors and interests within a heterosexual dating environment (Hill and Lynch, 1983). Thus, it is unclear whether boys would still be more restricted in their behavior than girls and generally how prescriptive stereotypes may change or emerge for adolescents and young adults.

On the other side of the age range, research has not focused on prescriptive gender stereotypes in the elderly. There is some evidence that descriptive gender stereotypes become more similar for elderly targets, in part because men's attributes become less masculine (Kite et al., 1991; DeArmond et al., 2006; Thompson, 2006). Conversely, other evidence shows that when compared to old women, older men are still seen as more competent, higher in autonomy, and less dependent (Canetto et al., 1995), demonstrating the continued existence of gender stereotypes. However, most of the research on aging stereotypes measures the negativity of the stereotypes (e.g., Hummert et al., 1995; Laditka et al., 2004) and not whether they are gendered. Thus, researchers have not addressed prescriptive stereotypes in the elderly or compared these to stereotypes of young adult or middle-aged men and women. Perhaps elderly men have less pressure to demonstrate their manhood and provide for a family, and thus their restrictions lessen, making violations of gender roles less severe than for younger individuals.

### Current research

In 3 studies, the current research measured prescriptive and descriptive gender stereotypes for various age groups, including children, adults, and the elderly. In all studies, participants rated how desirable and typical it was for different target groups to possess a list of characteristics. The list of characteristics included a variety of traits and behaviors, many of which have not been used in past research on adult stereotypes, to cover the types of behaviors that may be more relevant to childhood. For example, research on the parental treatment of boys vs. girls demonstrated higher levels of pressure for gendered interests and activities rather than traits (e.g., Lytton and Romney, 1991).

Through this method, the current research attempts to measure prescriptive gender stereotypes of toddlers, elementary-aged children, adolescents, young adults, adults, and the elderly to compare the content and strength of these stereotypes and answer several questions. In particular, assuming that gender stereotypes toward children and the elderly are also prescriptive in nature, current research addresses how both the content and magnitude of prescriptive gender stereotypes changes across age groups. Specifically, based on the emphasis on policing boys' behavior in childhood, one might expect that prescriptive stereotypes would be stronger for boys than adult men. Alternatively, these stereotypes may remain strong across age groups. Conversely, however, prescriptive feminine stereotypes may start weaker for girls and increase with age. Because descriptive stereotypes were also measured, prescriptive stereotypes can be compared to the typicality of each characteristics in males and females. Secondly, the research compares the number and magnitude of PPS and NPS for males and females within each age group to answer the question of whether males are more restricted than females in their behavior. Participants also answered a direct question comparing the desirability of stereotype violating behavior in males vs. females. Research suggests greater restrictions for males are likely for children, but the difference in strength and magnitude of prescriptive gender stereotypes has not been directly tested for specific age groups of children or for adult or elderly stereotypes.

## Method

### Participants

Student participants in Studies 1 and 2 took part in a laboratory setting for course credit. In Study 1 (*n* = 137), participants were 64.2% women; the mean age was 18.73 years (*SD* = 1.07); 72.3% were White/Caucasian, 16.8% Hispanic/Latino, 11.7% Asian, 5.1% Black/African American, and 6.6% other or unreported (in all studies participants could select as many racial groups as apply). In Study 2 (*n* = 91), participants were 65.9% women; the mean age was 19.10 years (*SD* = 1.97); 76.9% were White/Caucasian, 15.4% Asian, 12.1% Hispanic/Latino, 2.2% African American, and 8.8% other or unreported.

In Study 3 (*n* = 120), participants recruited through Amazon's Mechanical Turk (MTurk; see Buhrmester et al., 2011; Mason and Suri, 2012) participated for $0.30 for a 15-min survey. Participants were 59.3% women; the mean age was 38.17 years (*SD* = 13.67); 70.8% were White/Caucasian, 7.5% Hispanic/Latino, 6.7% Black/African American, 5.0% Asian, and 4.1% other or unreported.

### Procedure and designs

All procedures were approved by the USD Institutional Review Board and all materials are available upon request. Participants in Studies 1 and 2 gave written informed consent, but participants in Study 3 indicated their informed consent online as a waiver of written consent was obtained from the IRB. Participants in all three studies rated the prescriptive and/or descriptive stereotypes of 3–6 groups of boys/men and/or girls/women. In Study 1, each participant rated 3 target groups of either males or females of different ages in a 3 (target age: elementary school, adults, elderly) × 2 (target sex: male, female) × 2 (stereotype rating: prescriptive, descriptive) mixed-model design, with target age and stereotype rating as within-subjects. In Study 2, targets were expanded to more age groups and participants rated 2 target groups of males and females of the same age in a 5 (target age: toddlers, elementary-aged, adolescent, young adult, adult) × 2 (target sex: male, female) × 2 (stereotype rating: prescriptive, descriptive) mixed-model design, with target sex and stereotype rating as within-subjects. In Study 3, the sample was broadened to community participants, who rated 6 groups of males or females of various ages in a 6 (target age: toddlers, elementary-aged, adolescent, young adult, adult, elderly) × 2 (target sex: male, female) × 2 (stereotype rating: prescriptive, descriptive) mixed-model design, with target age as within-subjects. In all studies, the levels of the within-subject variable were presented in a random order. Target age was designated with a label and a corresponding age group: toddlers (~2–5 years old), elementary-aged children (~5–12 years old), adolescents (~12–18 years old), young adults (~18–30 years old), adults (~30–50 years old), the elderly (over ~65 years old). See Table 1 for a comparison of study designs.

**Table 1:** Comparison of the three Studies' methods.

	**Study 1**	**Study 2**	**Study 3**
Target age groups			
Toddlers (~2–5 years old)		X	X
Elementary-aged (~5–12 years old)	X	X	X
Adolescent (~12–18 years old)		X	X
Young adult (~18–30 years old)		X	X
Adult (~30–50 years old)	X	X	X
Elderly (over ~65 years old)	X		X
Design			
Target age	Within-subjects	Between-subjects	Within-subjects
Target sex	Between-subjects	Within-subjects	Between-subjects
Stereotype rating	Within-subjects	Within-subjects	Between-subjects

The instructions stated that the survey asked about the desirability of characteristics for males and females of different age groups. In Studies 1 and 2, prescriptive stereotype ratings were presented first, then the comparison of prescriptive stereotypes, and finally the descriptive ratings. To circumvent social desirability pressures, the instructions pointed out that the researchers were not interested in personal opinions but judgments of how society evaluates these characteristics for males and females of different age groups. Participants were then thanked for their time and debriefed about the purpose of the study.

A sensitivity analysis in G^*^Power (Faul et al., 2007) demonstrated that this research was able to detect with 80% power a between-subjects target sex effect of *d* = 0.37 in Study 1, a within-subjects target sex effect of *d* between 0.53 and 0.50 (with *n* between 17 and 19 per target age condition) in Study 2, and a between-subjects target sex effect of *d* = 0.55 for prescriptive stereotypes and *d* = 0.56 for prescriptive stereotypes in Study 3. Thus, with a cut-off of *d* = 0.40 to define a prescriptive stereotype, these studies had acceptable power to detect effects of larger magnitudes, although results from near the cutoff should be taken with caution.

### Measures

#### Prescriptive stereotypes

In Studies 1 and 2 participants rated the characteristics of target groups in response to the question, “How DESIRABLE it is in American society for [elementary school boys (~5–12 years old)] to possess the following characteristics? That is, we want to know how [boys] SHOULD act” [emphasis in original]. In Study 3 the second sentence read, “That is, regardless of how boys actually act, we want to know how society thinks [elementary school boys] SHOULD act.” The scale ranged from 1 (*very undesirable*) to 9 (*very desirable*). This question is similar to the prescriptive stereotype question and response options from Prentice and Carranza (2002), who also used a bi-polar scale.

#### Descriptive stereotypes

In Studies 1 and 2 participants also rated the characteristics of target groups in response to the question, “Indicate how COMMON or TYPICAL each of the following characteristics is in [elementary school boys (~5–12 years old)] in American society. That is, we want to know how adult females USUALLY act” [emphasis in original]. In Study 3, the question asking about descriptive stereotypes read “How COMMON or TYPICAL is it in American society for [elementary school boys (~5–12 years old)] to possess the following characteristics? That is, we want to know how society thinks [boys] USUALLY act.” In all studies the scale ranged from 1 (*very atypical*) to 9 (*very typical*).

#### Characteristics

Both types of stereotypes were rated on 19–21 characteristics, created by grouping the traits from previous research (Martin, 1995; Prentice and Carranza, 2002; Rudman et al., 2012b) based on similarity, and adding some additional characteristics to cover a larger variety of traits and behaviors and include characteristics more applicable to children (e.g., shy, noisy, interests, play, and dress style). The full list of characteristics is given in Table 2.

**Table 2:** Characteristics rated for prescriptive and descriptive stereotypes.

**Characteristic**	**Trait grouping**
Agentic	Assertive, competitive, achievement-oriented, leadership ability
Communal	Nurturing, warm, sensitive, gentle
Dominant	Dominant, aggressive, arrogant, intimidating
Weak	Weak, insecure, yielding, easily frightened
Emotional	Emotional, moody, melodramatic
Intelligent	Intelligent, analytical, competent, rational
Independent	Independent, self-reliant, ambitious
Shy	Shy, reserved, nervous, soft-spoken
Active	Active, energetic, athletic
Likeable	Likeable, cheerful, enthusiastic
Helpful	Helpful, friendly, cooperative, dependable
Wholesome	Wholesome, polite, naïve
Rebellious	Rebellious, stubborn, angry, self-centered
Noisy	Noisy, boisterous, rambunctious
Sexually active	Sexually active, promiscuous
Masculine interests	Interested in things like science, math, technology, and mechanical objects
Masculine appearance	Wears blue, wears loose-fitting clothes, strong
Masculine toys^a^	Interested in playing with trucks, blocks, and rough-and-tumble games
Feminine interests	Interested in things like languages, arts, and helping others
Feminine appearance	Wears pink, wears tight-fitting clothes, dainty
Feminine toys^a^	Interested in playing with dolls, dress-up, and role-playing house

a *Used only in Studies 2 and 3*. *The trait groupings are the items used in the stereotype ratings and the characteristic represents the label for the overarching concept being measured. The list was displayed in a different order for each study*.

To make it easier for participants to rate groups of characteristics (instead of individual traits), participants were instructed to note that not all traits would apply equally across age groups, but within each list of characteristics some may apply more to some age groups than others. Participants were asked to think about the meaning of the overall list as they rated each group, instead of focusing only on 1 or 2 traits in the list. One benefit of grouping traits this way is that it allowed the characteristics to be more applicable across age groups. Participants may have focused on slightly different traits, but all of the traits on a list represented the overall concept being measured, allowing for a comparison of that concept across ages even thought it might manifest as different behaviors in different age groups. Thus, participants could apply that concept to a certain age group, instead of attempting to rate an individual trait that may or may not seem relevant to each age group.

#### Prescriptive comparisons

In Studies 1 and 2, participants were also asked to compare the desirability of behavior of males and females who are likely violating their prescriptive stereotypes. Specifically, in two questions, participants compared (a) males (of a certain age) acting communal to females (of the same age) acting agentic (PPS of the other sex) and (b) males (of a certain age) acting weak to females (of the same age) acting dominant (NPS for that sex). Communion, agency, weakness, and dominance were defined using the same lists of characteristic given in Table 2. The scale ranged from 1 (*considerably less desirable for males to act nurturing/weak*) to 7 (*considerably less desirable for females to act assertive/dominant*).

## Results and discussion

The raw data supporting the conclusions of this article can be requested from the author. Effect sizes for both prescriptive and descriptive stereotypes are the standardized difference between the relevant conditions, or Cohen's *d*. I corrected the small-sample bias in estimates of *d* using the conversion to Hedges' *g*, but refer to the effect sizes as *d*. In Study 1 and 3, effect sizes were calculated by dividing the difference in ratings for male and female targets at each of the different age groups by the pooled standard deviation. In Study 2, where target sex was within-subjects, effect sizes were calculated by dividing the difference in ratings by the average standard deviation, in order to facilitate the meta-analysis across studies (see Lakens, 2013). These effect sizes were then meta-analyzed using fixed-effects across the three studies, when the same age group was rated. A fixed-effects rather than random-effects meta-analysis was more appropriate because the studies had nearly identical measures and the sample of studies was too small to yield a reliable estimate of the between-study variability needed in random-effects computations (see Borenstein et al., 2009).

### Prescriptive stereotypes

Table 3 provides the effect sizes in the meta-analysis of prescriptive stereotypes (see the Supplementary Tables for effects for each study separately). As defined by Rudman et al. (2012b), prescriptive stereotypes were defined as traits displaying a sex difference of *d* > 0.40 and an average rating as desirable (>6 for PPS) or undesirable (<4 for NPS) for males or females. These two criteria mean that a large difference between the desirability of the characteristic between males and females does not necessarily classify as a stereotype if it is not also highly desirable or undesirable for one sex. Based on these criteria, PPS and NPS for males and females are designated in Table 3. To facilitate comparisons across age groups, the bottom rows of Table 3 report the number of characteristics that meet the criteria to be considered as PPS and NPS and the average effect size for these PPS and NPS.

**Table 3:** Meta-analyzed prescriptive stereotypes (*d*) by target age.

**Characteristic**	**Toddlers**			**Elementary-school age**			**Adolescents**			**Young adults**			**Adults**			**Elderly**		
	***d***	***M***	***F***	***d***	***M***	***F***	***d***	***M***	***F***	***d***	***M***	***F***	***d***	***M***	***F***	***d***	***M***	***F***
Agentic	0.45			**0.47**	+		**0.76**	+		**0.89**	+		**1.09**	+		**0.59**	+	
Communal	−**0.45**		+	−**0.85**		+	−**0.93**		+	−**1.23**		+	−**1.01**		+	−**0.63**		+
Dominant	0.16			**0.66**		–	**0.63**		–	**0.85**		–	**0.86**		–	0.26		
Weak	−0.33			−**0.70**	–		−**0.72**	–		−**0.68**	–		−**0.85**	–		−**0.58**	–	
Emotional	−0.33			−**0.51**	–		−**0.69**	–		−**0.69**	–		−**0.48**	–		−0.07		
Intelligent	−0.08			−0.04			0.33			**0.53**	+		**0.68**	+		**0.54**	+	
Independent	0.17			0.37			**0.56**	+		**0.55**	+		**0.97**	+		0.26		
Shy	−0.42			−**0.93**	–		−**0.84**	–		−**0.95**	–		−**0.97**	–		−0.46		
Active	0.12			**0.44**	+		**0.75**	+		0.33			**0.43**	+		0.11		
Likeable	−0.27			−0.31			−**0.82**		+	−0.24			−0.23			−0.05		
Helpful	−0.23			−0.27			−0.38			−0.33			−0.16			−0.23		
Wholesome	−0.23			−**0.47**		+	−**0.52**		+	−**0.49**		+	−0.29			−0.23		
Rebellious	0.30			0.22			0.12			**0.54**		–	0.35			0.16		
Noisy	0.27			**0.43**		–	**0.58**		–	**0.64**		–	**0.57**		–	0.00		
Sexually active	−0.00			0.05			**0.53**		–	0.40			**0.58**	+		0.36		
Masculine interests	0.23			**0.56**	+		**0.73**	+		**0.70**	+		**1.04**	+		**0.65**	+	
Masculine appearance	**1.36**	+		**1.36**	+		**1.40**	+		**1.33**	+		**1.52**	+		0.37		
Masculine toys	**1.49**	+		**1.53**	+		**0.96**		–	**0.86**		–	**0.50**		–	−0.15		
Feminine interests	−0.38			−**0.47**		+	−**0.86**		+	−**0.66**		+	−0.34			0.03		
Feminine appearance	−**2.04**	–	+	−**2.02**	–	+	−**2.23**	–	+	−**2.25**	–	+	−**2.13**	–		−**0.46**	–	
Feminine toys	−**2.21**	–	+	−**2.63**	–	+	−**1.53**	–		−**1.38**	–		−**0.93**	–		−**0.82**	–	
Count PPS		2	3		5	5		5	5		5	4		7	1		3	1
Count NPS		2	0		5	2		5	4		5	4		5	3		3	0
Average PPS		1.43	1.57		0.87	1.29		0.84	1.07		0.80	1.16		0.90	1.01		0.59	0.63
Average NPS		2.12	0.00		1.36	0.54		1.20	0.68		1.19	0.72		1.07	0.65		0.62	0.00

*Positive d-values reflect males were rated higher on that characteristic and negative d-values reflect women were rated higher on that characteristic. d-values in bold meet the criteria to be labeled a positive prescriptive or negative proscriptive stereotype for men or women, with a + indicating a prescription and a – indicating a proscription. Count PPS and Count NPS represent the number of characteristics that meet the criteria to be labeled prescriptive stereotypes and Average PPS and Average NPS are the average effect size of the characteristics that meet the criteria for that age group*.

It is clear from these data that prescriptive gender stereotypes exist across age groups, satisfying the assumption that prescriptive stereotypes are relevant for each age group. Thus, the data are described in relation to two questions: (a) comparing the content and magnitude of prescriptive gender stereotypes across age groups and (b) comparing the magnitude of PPS and NPS for males and females within each age group.

#### Comparisons across target age

Toddlers had very few prescriptive stereotypes, and with the exception of being communal for girls, their stereotypes were not about traits but physical appearance and toys. Toddler boys had both strong PPS to have a masculine appearance and play with masculine toys and NPS to avoid having a feminine appearance or playing with feminine toys. Girls had strong PPS to have a feminine appearance and play with feminine toys as well as a weaker PPS to be communal. Although these prescriptive stereotypes were strong, other trait-based stereotypes were much weaker, suggesting that people do not have gendered expectations of toddlers' traits—perhaps because their personalities are perceived as not yet formed and more malleable (e.g., Neel and Lassetter, 2015). People do, however, have strong prescriptions about how toddlers should look and what they should play with, contradicting Campenni's (1999) research showing that gender-appropriateness of toys for toddlers were less stereotypical than ratings for older children.

As early as elementary school, prescriptive gender stereotypes similar to those for adults emerged. The strongest stereotypes for school-aged children were again for physical appearance and behavior, with the same pattern as for toddlers. At this age, sex-typed interests also appeared as prescriptive stereotypes, where it was seen as desirable for boys to be interested in math and science and girls to be interested in language and arts—but it is important to note that opposite sex-typed interests did not meet the criteria for proscriptive stereotypes. Trait stereotypes also met the criterion for elementary school-aged children: It was desirable for boys to be agentic and active and avoid being shy, weak, or emotional. Girls, on the other hand, should be communal as well as wholesome and avoid being dominant or noisy. These prescriptive stereotypes are very similar to those found by Martin (1995) for 4–7 year old children, including agency, interest in mechanical objects, rough play and avoiding weakness for boys and communal traits and avoiding noise for girls. The proscription of shyness for boys of this age group is also consistent with Doey et al's. (2014) analysis of the social (in)acceptability of shyness for school-aged boys. Martin (1995) did label independence as a desirable trait for boys (which did not meet the criteria for a prescriptive stereotype until adolescents in these data) and being neat, well-mannered, and helpful around the house for girls, which were not directly measured in the current data.

Stronger prescriptive gender stereotypes may emerge in elementary school-aged children, compared to toddlers, because by this age people believe that counterstereotypical behavior is predictive of adult counterstereotypical behaviors (Sandnabba and Ahlberg, 1999), and so prescriptive stereotypes become relevant in order to pressure normative behavior. Thus, people appear to believe that elementary-aged children are no longer considered as malleable in their personality as toddlers. Conversely, there was no evidence for the idea that stereotypes for children would be stronger than stereotypes of adults—if anything, they were slightly weaker, although not by much.

Trait prescriptive stereotypes of male and female adolescents were intensified slightly compared to younger children, but not to a high degree and the average prescriptive stereotypes were not different in magnitude from younger children. These stereotypes were also not much different than adult stereotypes. Thus, there is not a lot of support for the idea that adolescence highlights gender differences and intensifies prescriptions based on the magnitude of the stereotypes.

There were some changes in the content of the stereotypes in adolescence and young adulthood, however. Starting in adolescence, PPS for toy/play behavior fell away for both males and females, although NPS to avoid opposite sex-typed toys remained with females picking up the admonition to avoid masculine toys. Stereotypes for physical appearance also remained, at about the same magnitude as for children. PPS for males to be agentic and independent as well as be interested in math and science increased from adolescence into adulthood, but the stereotype for males to be active peaked in adolescence. These PPS are now similar in magnitude to NPS for males to avoid being shy, weak, or emotional. Young adulthood brings a new PPS for males to be intelligent, which remains with age.

For females, adolescence bought a PPS to be likeable and a NPS to be sexually active and young adulthood a NPS for rebelliousness, but none of these stereotypes met the criteria for a stereotype in any other age group. PPS for girls and women to be communal grew with age and peaked in young adulthood, and NPS to avoid dominance grew into adulthood as well. The strongest prescriptive stereotypes for adolescent girls through adult women were to have a feminine appearance and be communal and avoid dominance and masculine toys.

These results replicated previous research on prescriptive stereotypes for adults (Prentice and Carranza, 2002; Rudman et al., 2012b), showing that women should be communal and avoid being dominant and men should be agentic and independent but avoid being weak and emotional. Adult prescriptive stereotypes were expanded in the current study by including more characteristics: Women should also have a feminine appearance and be interested in languages/arts, and avoid having a masculine appearance and being sexually active or noisy. Men should also have a masculine appearance, be interested in science/math/technology/mechanical objects, and be sexually active, but avoid being shy and appearing feminine. Adult men were also supposed to be sexually active, compared to women.

Stereotypes for the elderly were weaker for both men and women. Men were still supposed to have masculine interests, be agentic, and be intelligent as well as avoid feminine toys, appearing feminine, and weakness, but these stereotypes were weaker than those for adults from 30 to 50 years old. For elderly women, all stereotypes fell away except for a PPS to be communal, which was also weaker than for other age groups (excepting toddlers). These results are consistent with the findings that descriptive gender stereotypes weaken for elderly targets (e.g., DeArmond et al., 2006; Thompson, 2006). These stereotypes were also inconsistent across studies (see Supplementary Tables), suggesting that prescriptive gender stereotypes may be less relevant to older age groups.

Overall, these results demonstrated that the content and magnitude of prescriptive stereotypes do change for different age groups, focusing on activities and appearance at the youngest ages studied here, with trait stereotypes increasing for elementary-aged children and continuing through adulthood. There was not much evidence for an intensification of prescriptive gender stereotypes for adolescents, as these stereotypes were similar to both the elementary and young adult age groups. Stereotypes then waned for elderly targets, supporting the notion that prescriptive gender stereotypes also weaken with age.

#### Comparison of male vs. female stereotypes

One test of the question of whether males' behavior is more restricted than females' behavior depends on the number and magnitude of the PPS and NPS in each age group. Based on the data counting and averaging prescriptive stereotypes of males and females of each age group presented in Table 3, the stereotypes were more restrictive for males than females at nearly every age group. Although toddlers had few prescriptive stereotypes, the ones that did exist demonstrated that toddler boys had both strong PPS and NPS, whereas girls had only strong PPS but no strong NPS to avoid masculine things. From elementary-aged through adults, females gained weak NPS and the magnitude of male PPS and NPS decreased slightly, but overall the same pattern held. Even though stereotypes for the elderly are weaker for both males and females, the prescriptive stereotypes were still more numerous and stronger for men than women.

In nearly every age group (except the elderly), the average NPS were larger than PPS for males, suggesting that males are directed more based on what they should *not* do rather than what they should do. Conversely, female PPS stereotypes were stronger than female NPS and male PPS, thus females are directed more based on what they *should* do rather than what they should not do. Thus, the stronger pressure on males to conform to gender stereotypes focuses on telling boys and men behaviors to avoid. This idea is interesting in relation to precarious manhood, which suggests that men's status as a man is easily lost—especially if they display feminine behaviors (Vandello and Bosson, 2013) that in this research made up NPS for males.

A second test of this question of greater restrictions for males involves the prescriptive comparison bi-polar questions that directly asked participants whether it was less desirable for males or females to violate stereotypes. These questions were identical in Studies 1 and 2 (but omitted in Study 3), and the means are presented in Table 4. It is worth noting that in the current study agency did not meet the criterion for a NPS for females and communion did not meet the criterion for a NPS for males. However these characteristics were PPS for the other sex, and this question is labeled as positive violations because it describes males and females acting in ways prescribed to the other sex. Weakness and dominance were proscribed behaviors for males and females, respectively, and thus these are labeled negative violations because for males to act weak and females to act dominant violates NPS.

**Table 4:** Means and standard deviations for comparisons for desirability of violating prescriptive stereotypes by target age.

	**Toddlers**		**Elementary-aged**		**Adolescents**		**Young adult**		**Adult**		**Elderly**	
	***M***	***SD***	***M***	***SD***	***M***	***SD***	***M***	***SD***	***M***	***SD***	***M***	***SD***
**POSITIVE VIOLATIONS**												
Study 1			3.64_a_^*^	1.58					4.26_b_	1.76	4.75_c_^*^	1.29
Study 2	4.37_a_^*^	1.41	3.37_bc_^*^	1.49	3.04_b_^*^	1.50	3.59_c_^*^	1.65	3.93_d_	1.37		
**NEGATIVE VIOLATIONS**												
Study 1			3.84_a_	1.52					2.84_b_^*^	1.69	4.15_c_	1.55
Study 2	4.30_a_	1.45	3.76_b_	1.52	2.82_c_^*^	1.41	2.85_c_^*^	1.61	3.41_b_^*^	1.56		

Means with the different subscripts differed by p < 0.05. Means with

* *were significantly different from the midpoint of the scale (4) at p < 0.05. Means lower than 4 indicate it was less desirable for males than females to violate the stereotype, means above 4 indicate it was less desirable for females than males to violate the stereotype*.

Most of the means were different from the midpoint of the scale (4), except for positive violations for adults and negative violations for elementary-aged, elderly (in Study 1), and toddlers (in Study 2). Repeated measures analysis of variance (ANOVA) on the positive and negative violations demonstrated that ratings varied by target age for positive violations in Study 1, *F*_(2, 256)_ = 21.34, *p* < 0.001, partial η^2^ = 0.14, and Study 2, *F*_(4, 360)_ = 14.09, *p* < 0.001, partial η^2^ = 0.14, and for negative violations in Study 1, *F*_(2, 258)_ = 36.73, *p* < 0.001, partial η^2^ = 0.22, and Study 2, *F*_(4, 360)_ = 22.09, *p* < 0.001, partial η^2^ = 0.20. Contrasts showed that for positive violations, it was less desirable for males to be communal than females to be agentic for adolescents, elementary-aged, and young adults but less desirable for females to be agentic than males to be communal in toddlers and the elderly. For negative violations, it was less desirable for males to be weak than females to be dominant for adolescents, young adults, and adults, and in no cases was it less desirable for females to be dominant than for males to be weak.

These results support the notion that males' behavior is more restricted than females even when asking people directly to compare the behaviors of males and females. Although toddlers and the elderly were exempt from these restrictions, there was greater concern, compared to females being agentic or dominant, that (a) elementary-aged boys should not be communal, (b) adolescent boys and young adult men should be not be communal or weak, and (c) adult men should not be weak. A greater emphasis on males' than females' prescriptive violations in these questions was strongest for adolescents, supporting the idea that these concerns more strongly emerge at puberty, even though the overall magnitude of prescriptive stereotypes were not strongest for adolescents. Interestingly, concerns for the positive violations of the elderly reverse, such that it was more concerning if females behave agentically than if males behave communally, consistent with the idea that male stereotypes evolve to include more communal elements in the elderly. Thus, these data that required participants to directly compare the violation of stereotypes for males and females supported the conclusion that males are more restricted in their behavior from elementary school to adulthood.

#### Prescriptive stereotype summary

In sum, these findings demonstrated the applicability of prescriptive stereotypes to different age groups, but also their variation depending on the age of the target group. The largest stereotypes for toddlers and elementary-aged youth were for girls to have and for boys to avoid a feminine appearance and playing with feminine toys. Prescriptive stereotypes for very young boys and girls were focused on appearance and play behaviors, and were especially proscriptive for boys—telling them more what not to do than what to do. Trait stereotypes appeared for elementary school-aged children, and the prescriptions for the usual suspects of communion, agency, dominance, and weakness remained into adulthood. Stereotypes for the elderly were then again minimized, demonstrating that people hold elderly men and women to few standards of gendered behavior, although elderly men still had more prescriptive stereotypes than elderly women. Overall, it does appear that males received more pressure in the form of prescriptive stereotypes, especially NPS about what not to do, across all age groups and especially for toddlers.

### Descriptive stereotypes

Table 5 displays the average effect size across the three studies in the meta-analysis of descriptive stereotypes. The Supplementary Tables show the effect sizes for each study separately. Similar to Martin (1995), the effect sizes were often larger for descriptive than prescriptive stereotypes not only for children but for most age groups. Using criterion of *d* > 0.40 (similar to the prescriptive stereotype criterion) to qualify as a descriptive stereotype, 98 out of 126 (77.8%) effects over all age groups qualify as descriptive stereotypes. Thus, males and females were often rated as typically different even when the behavior was not prescribed for one sex over the other. However, descriptive stereotypes were highly correlated with prescriptive stereotypes for toddlers, *r*_(19)_ = 0.95, *p* < 0.001, elementary-aged, *r*_(19)_ = 0.97, *p* < 0.001, adolescents, *r*_(19)_ = 0.94, *p* < 0.001, young adults, *r*_(19)_ = 0.94, *p* < 0.001, adults, *r*_(19)_ = 0.95, *p* < 0.001, and the elderly, *r*_(19)_ = 0.77, *p* < 0.001. Thus, prescriptive and descriptive stereotypes aligned, although these high correlations may be an outcome of having the same participants rate both desirable and typical behaviors in Studies 1 and 2.

**Table 5:** Meta-analyzed descriptive stereotypes (*d*) by target age.

**Characteristic**	**Toddlers**	**Elementary-school age**	**Adolescents**	**Young adults**	**Adults**	**Elderly**
Agentic	0.40	0.35	0.90	0.63	0.93	0.83
Communal	−0.82	−0.97	−0.89	−0.91	−1.32	−0.88
Dominant	0.95	0.97	1.25	1.07	1.40	0.83
Weak	−0.37	−0.75	−0.84	−0.41	−0.93	−0.53
Emotional	−0.37	−0.82	−1.00	−0.83	−1.30	0.02
Intelligent	−0.39	−0.43	−0.17	0.28	0.48	0.60
Independent	0.25	0.11	0.17	0.03	0.72	0.31
Shy	−0.34	−0.91	−0.56	−0.46	−1.03	−0.46
Active	0.25	0.67	0.48	0.07	0.47	0.24
Likeable	−0.44	−0.30	−0.58	−0.68	−0.31	−0.29
Helpful	−0.47	−0.61	−0.43	−0.51	−0.65	−0.45
Wholesome	−0.63	−0.67	−0.65	−0.25	−0.56	−0.51
Rebellious	0.32	0.74	0.35	0.78	0.60	0.69
Noisy	0.44	0.84	0.57	0.54	0.41	0.39
Sexually active	0.30	0.34	0.48	0.64	0.50	0.61
Masculine interests	0.71	0.64	0.73	0.57	1.17	1.14
Masculine appearance	0.99	1.46	1.20	1.14	1.49	0.67
Masculine toys	1.45	1.68	1.22	0.85	0.75	0.79
Feminine interests	−0.16	−0.90	−1.10	−0.73	−0.76	−0.04
Feminine appearance	−2.08	−2.35	−1.58	−2.03	−1.87	−0.64
Feminine toys	−2.11	−2.51	−1.05	−0.69	−0.49	0.24

*Positive d-values reflect males were rated higher on that characteristic and negative d-values reflect females were rated higher on that characteristic*.

### Limitations and future research

It is important to note that this research was conducted with majority White samples from the United States. The predominately White samples likely used White targets as their reference group, since target race was not specified. Thus, caution should be used when extrapolating the results to participants or targets of other racial groups. Previous research has demonstrated that that descriptive stereotypes of men and women are more similar to stereotypes of White men and White women than to gender stereotypes of other racial groups (Ghavami and Peplau, 2013) and Blacks are seen as more masculine and Asians as more feminine than Whites (Galinsky et al., 2013). There is also reason to suspect that prescriptive gender stereotypes may vary by race, as Black female leaders do not experience backlash for being dominant (Livingston et al., 2012). Thus, it is important to acknowledge the current results describe stereotypes of Whites for Whites, but more research will be needed to know if other racial groups show similar prescriptive gender stereotypes for different age groups and if men of other racial groups are more restricted in their behavior than women.

In addition, the current ratings were all perceptions of adults (college students or older) of various age groups, from toddlers to the elderly. Missing are ratings of each age group of its own stereotypes (e.g., toddlers of toddlers; adolescents of adolescents; the elderly of the elderly). Suggesting similarity in prescriptive stereotypes across participant age groups, previous research demonstrated that children's reactions to norm violators (e.g., Smetana, 1986; Levy et al., 1995) show the same pattern of greater disapproval of counterstereotypical behavior from boys than girls that adults demonstrate in other studies. In addition, Powlishta (2000) found that children's and adults' descriptive stereotypes of child and adult targets were quite similar, although the difference between ratings of males and females on femininity was weaker for child than adult participants. Descriptive stereotypes of the elderly were also weaker for elderly respondents than middle-age or young respondents (Hummert et al., 1995). It is unknown whether similar effects of participant age would occur for prescriptive stereotypes, which might be conceptually more difficult for children to understand as they designate desirable behavior rather than actual behavior. Stereotypes of one's own age group would be interesting to study, but with the current data I was interested in whether adults view different age groups differently. The stereotypes adults hold about children impact how children behave through gender role socialization, modeling, and direct tutelage (Witt, 1997; Bussey and Bandura, 2004). Adults' beliefs about adolescents can also be important, as parents' stereotypical beliefs about adolescents' focus on peers and social concerns impacted parents' perceptions and their child's behavior (Jacobs et al., 2005). Thus, parental beliefs about gender stereotypes can influence their children's gender role behavior, so understanding adults' views of children is important. Future research could assess whether parental status matters to these views, to see if greater familiarity with children or adolescents changes adults' views of prescriptive gender stereotypes.

The current research also did not assess possible reasons for the differences in prescriptive stereotypes across age groups. For example, the research did not attempt to measure the impact of stereotype violations on status, manhood, or perceived sexual orientation, which are all possible mechanisms for the policing of boys and men in terms of what they are not supposed to do. It may be the case that these mechanisms vary across age groups. The smaller prescriptive stereotypes in toddlers may be due greater perceived malleability in personality and trait characteristics, and behaviors of younger children may not speak as directly to sexual orientation (see McCreary, 1994). In addition, if these concerns are reduced or removed for the elderly, this may help to explain the reduced size of prescriptive gender stereotypes in this age group. Future research should continue to address these issues across a wide variety of age groups.

The meta-analytic results presented here average across three studies with different research designs. However, it is important to note that Study 2 had larger effect sizes (see Supplementary Tables), most likely because target sex was within-subjects, encouraging participants to draw sharper distinctions between the male and female groups. These target contrast effects have occurred in other research. For example, Thompson (2006) found that old men were rated as more masculine and less feminine when compared to old women than when compared to young men. Participants in the current research rated the targets in a random order by age, minimizing any one specific age comparison when averaging across participants, but stereotypes may also differ depending on the presentation order of age groups. Thus, the size of the stereotypes may depend on the research design used to capture them.

### Implications

Because prescriptive stereotypes exist across age groups, the mechanism causing the negative reactions and backlash to counterstereotypical behavior may be the same for both children and adults—a violation of prescriptive stereotypes. However, different types of behavior would violate prescriptive stereotypes in adults and children, based on the specific content and magnitude of these stereotypes. For example, negative reactions to children might focus more on violations of physical appearance or play behaviors, rather than traits, whereas reactions to adolescents and adults could result from violations of both trait and appearance prescriptive stereotypes. Future research should address prescriptive stereotypes as a mechanism for negative reactions to children, adults, and the elderly who display counterstereotypical behaviors. Backlash could also vary with perceiver's ideology—non-traditional participants might see stereotype violations as a positive rather than a negative event (see Gaunt, 2013).

## Conclusions

The current findings demonstrated the applicability of prescriptive stereotypes to different age groups, from toddlers to the elderly, and presented their content and magnitude. All age groups had prescriptive stereotypes, although the content and magnitude of those stereotypes varied across age groups. Prescriptive stereotypes for toddlers contained elements of play and appearance, whereas trait stereotypes appeared starting for elementary-aged children. Prescriptive stereotypes for the elderly were minimized, suggesting less pressure to conform to expectations. Prescriptions for males focused on NPS that admonish what not to do, whereas females' stronger PPS focused on what girls and women are supposed to do. Thus, overall, males' behavior was more restrictive based on these stereotypes. The current research describes the current state of prescriptive gender stereotypes for a variety of age groups, and the consequences of these stereotypes for socialization and backlash as well as how the stereotypes might differ across racial groups deserve further study.

## Ethics statement

These studies were carried out in accordance with the recommendations of ethical standards of the American Psychological Association. The protocol were approved by the University of San Diego's Institutional Review Board (IRB). Participants in Studies 1 and 2 gave written informed consent in accordance with the Declaration of Helsinki, but in Study 3 participants did not because a waiver of written consent was granted by the IRB. Instead, participants consented online before participating in the study.

## Author contributions

AK conceived, planned, and carried out the experiments, analyzed the data, interpreted the results, and wrote the manuscript.

### Conflict of interest statement

The author declares that the research was conducted in the absence of any commercial or financial relationships that could be construed as a potential conflict of interest.
