# Supplementary material for: Comparing Prescriptive and Descriptive Gender Stereotypes About Children, Adults, and the Elderly
Source: Front Psychol. 2018 Jun 26;9:1086. doi: 10.3389/fpsyg.2018.01086 (PMC6028777; doi:10.3389/fpsyg.2018.01086)
Supplement: Supplementary file 1 [file Table_1.pdf]

Supplementary Material

Prescriptive and Descriptive Gender Stereotypes about Children, Adults, and the Elderly

Anne M. Koenig*

*** Correspondence: akoenig@sandiego.edu**

Tables S1-S4 present the effect sizes for prescriptive and descriptive stereotypes in each study separately (roughly sorted by size) and the mean desirability rating for men and women, only for the characteristics that met the criteria for a prescriptive or proscriptive stereotype (similar to tables from Rudman et al., 2012).

Table S1

Prescriptive Stereotypes, Mean Desirability Ratings, and Descriptive Stereotypes for Males and Females by Age Group in Study 1

	Prescriptive *d*	Male *M*	Female *M*	Descriptive *d*	Prescriptive *d*	Male *M*	Female *M*	Descriptive *d*	Prescriptive *d*	Male *M*	Female *M*	Descriptive *d*
	Elementary-aged				Adults				Elderly			
					Males' prescriptions							
Masculine appearance	1.36	7.30	4.82	1.60	1.84	7.61	4.62	1.98	0.59	6.72	5.69	0.99
Masculine interests	0.72	7.52	6.19	0.77	1.17	7.73	5.82	1.04	0.72	6.82	5.50	1.24
Agentic	0.51	7.22	6.18	0.30	0.99	8.39	6.84	0.87	0.74	6.82	5.43	1.01
Independent	--	--	--	--	0.94	8.39	7.03	0.93	--	--	--	--
Intelligent	--	--	--	--	0.74	8.65	7.84	0.56	0.69	8.06	6.87	0.85
Sexually active	--	--	--	--	0.58	6.39	5.12	0.41	--	--	--	--
Active	0.45	8.36	7.65	0.89	0.56	8.05	7.32	0.38	--	--	--	--
					Females' prescriptions							
Communal	-0.73	6.08	7.49	-1.09	-0.92	7.15	8.43	-1.32	-0.64	7.94	8.71	-1.13

	Prescriptive *d*	Male *M*	Female *M*	Descriptive *d*	Prescriptive *d*	Male *M*	Female *M*	Descriptive *d*	Prescriptive *d*	Male *M*	Female *M*	Descriptive *d*
	Elementary-aged				Adults				Elderly			
Feminine appearance	-1.96	2.49	6.09	-2.65	--	--	--	--	--			
Wholesome	--	--	--	--	--	--	--	--	-0.45	5.96	6.88	-0.57
Males' proscriptions												
Feminine appearance	1.96	2.49	6.09	-2.65	1.95	2.32	5.97	-2.10	--	--	--	--
Weak	0.76	2.83	4.28	-0.91	0.92	1.85	3.15	-1.21	0.68	3.27	4.49	-0.98
Shy	1.12	3.82	5.60	-1.19	0.89	3.29	4.66	-1.19	--	--	--	--
Emotional	0.46	2.90	3.75	-0.79	0.43	2.46	3.21	-1.64	--	--	--	--
Females' proscriptions												
Dominant	-0.56	3.72	2.56	0.95	-0.84	4.85	2.96	1.27	--	--	--	--
Noisy	--	--	--	--	-0.46	3.15	2.46	0.26	--	--	--	--
Sexually active	--	--	--	--	--	--	--	--	-0.47	3.25	2.35	0.68

Note. Positive *d* scores reflect stronger prescriptions, proscriptions, or descriptive stereotypes for males than females. Negative *d* scores reflect stronger prescriptions, proscriptions, or descriptive stereotypes for females than males.

Table 2

Prescriptive Stereotypes, Mean Desirability Ratings, and Descriptive Stereotypes for Males and Females by Age Group in Study 2

	Pres *d*	M *M*	F *M*	Des *d*	Pres *d*	M *M*	F *M*	Des *d*	Pres *d*	M *M*	F *M*	Des *d*	Pres *d*	M *M*	F *M*	Des *d*	Pres *d*	M *M*	F *M*	Des *d*
	Toddlers				Elementary School-Aged				Adolescents				Young Adults				Adults			
Males' prescriptions																				
Masculine toys	2.88	7.47	3.63	3.14	1.99	7.72	4.06	5.30	1.80	7.22	3.22	2.51	1.67	6.59	2.76	1.28	--	--	--	--
Masculine appearance	2.66	7.32	3.58	2.80	2.15	7.50	3.83	5.72	2.56	7.61	3.5	2.69	2.44	7.29	3.41	2.17	2.76	7.95	3.95	3.08
Active	1.22	7.84	6.42	0.91	1.21	8.56	7.44	1.38	1.71	8.67	6.72	1.30	0.82	8.29	7.41	0.68	1.08	8.16	6.89	1.13
Agentic	0.81	7.00	5.74	1.13	0.56	7.94	7.17	0.67	1.60	8.28	5.89	1.55	1.25	8.47	6.47	1.12	2.82	8.74	6.00	2.04
Dominant	--	--	--	--	1.58	6.11	3.06	3.12	--	--	--	--	--	--	--	--	3.05	6.42	2.26	2.48
Independent	--	--	--	--	1.14	7.94	6.28	0.69	0.99	8.17	6.89	0.76	0.93	8.24	7.00	0.72	2.24	8.74	6.42	1.46
Masculine interests	--	--	--	--	0.65	7.56	6.56	1.06	1.18	6.94	4.89	1.30	0.62	7.59	6.53	0.71	1.59	7.63	5.00	1.82
Intelligent	--	--	--	--	--	--	--	--	--	--	--	--	0.58	8.35	7.65	0.36	1.02	8.68	7.74	0.82
Sexually active	--	--	--	--	--	--	--	--	--	--	--	--	--	--	--	--	0.90	6.16	4.42	1.01
Females' prescriptions																				
Feminine appearance	-3.29	2.11	6.95	-5.03	-2.94	1.83	6.89	-9.62	-3.33	1.72	7.06	-6.05	-2.39	2.29	6.94	-5.15	-3.15	1.63	6.42	-3.42

	Pres *d*	M *M*	F *M*	Des *d*	Pres *d*	M *M*	F *M*	Des *d*	Pres *d*	M *M*	F *M*	Des *d*	Pres *d*	M *M*	F *M*	Des *d*	Pres *d*	M *M*	F *M*	Des *d*
	Toddlers				Elementary School-Aged				Adolescents				Young Adults				Adults			
Feminine toys	-2.35	2.79	7.26	-4.31	-3.59	2.22	7.67	-5.90	-3.75	1.28	6.67	-4.47	-2.80	1.53	6.35	-2.30	--	--	--	--
Communal	-0.85	6.63	7.89	-2.19	-1.65	5.39	8.11	-3.27	-2.19	5.06	8.33	-2.94	-2.57	5.94	8.71	-3.43	-1.99	5.32	8.47	-2.23
Feminine interests	-0.48	6.32	7.05	-0.29	-0.99	6.11	7.67	-1.43	-1.14	5.17	7.00	-1.65	-1.29	5.41	7.41	-1.21	-1.31	5.32	7.26	-1.81
Shy	--	--	--	--	-1.41	3.78	6.28	-2.18	--	--	--	--	--	--	--	--	--	--	--	--
Wholesome	--	--	--	--	-1.32	5.56	7.83	-1.59	-1.33	5.33	7.39	-1.63	-0.94	5.47	7.24	-1.59	-0.72	4.68	6.11	-1.25
Likeable	--	--	--	--	-0.55	7.67	8.22	-1.24	-1.31	7.17	8.61	-0.89	--	--	--	--	--	--	--	--
Helpful	--	--	--	--	-0.48	7.44	7.94	-1.39	-0.77	6.83	8.00	-1.03	-0.49	7.24	7.82	-0.76	-0.56	7.05	7.79	-1.57
Males' prescriptions																				
Feminine appearance	3.29	2.11	6.95	-5.03	2.94	1.83	6.89	-9.62	3.33	1.72	7.06	-6.05	2.39	2.29	6.94	-5.15	3.15	1.63	6.42	-3.42
Feminine toys	2.35	2.79	7.26	-4.31	3.59	2.22	7.67	-5.90	3.75	1.28	6.67	-4.47	2.80	1.53	6.35	-2.30	1.15	1.84	4.37	-1.31
Emotional	0.82	2.53	3.89	-0.92	1.43	2.44	4.89	-3.51	1.38	1.78	3.72	-2.84	1.02	2.00	3.82	-2.35	0.98	1.95	3.42	-3.00
Weak	0.55	2.84	3.74	-0.92	1.12	2.44	4.28	-2.43	1.99	1.5	4.17	-2.00	1.21	1.76	3.53	-1.06	1.48	1.58	3.53	-1.94
Shy	--	--	--	--	1.41	3.78	6.28	-2.18	1.39	2.72	5.00	-1.01	1.43	2.76	4.71	-1.93	1.72	3.16	5.26	-1.89

	Pres *d*	M *M*	F *M*	Des *d*	Pres *d*	M *M*	F *M*	Des *d*	Pres *d*	M *M*	F *M*	Des *d*	Pres *d*	M *M*	F *M*	Des *d*	Pres *d*	M *M*	F *M*	Des *d*
	Toddlers				Elementary School-Aged				Adolescents				Young Adults				Adults			
	Females' proscriptions																			
Masculine toys	-2.88	7.47	3.63	3.14	--	--	--	--	-1.80	7.22	3.22	2.51	-1.67	6.59	2.76	1.28	-0.87	4.79	2.84	1.05
Masculine appearance	-2.66	7.32	3.58	2.80	2.15	7.50	3.83	5.72	-2.56	7.61	3.5	2.69	-2.44	7.29	3.41	2.17	-2.76	7.95	3.95	3.08
Noisy	-0.80	4.00	2.74	1.38	0.77	4.67	3.06	1.51	-0.70	4.00	2.61	1.56	-1.19	3.65	1.94	2.25	-1.26	4.32	2.53	1.56
Dominant	--	--	--	--	1.58	6.11	3.06	3.12	-1.36	5.61	2.61	2.35	-1.48	5.82	2.71	3.14	-3.05	6.42	2.26	2.48
Rebellious	--	--	--	--	-0.55	3.89	3.11	1.86	--	--	--	--	-1.00	2.94	1.82	2.45	-1.10	3.58	2.05	1.38

Note. Pres = prescriptive, M = male, F = female, Des = descriptive. Positive *d* scores reflect stronger prescriptions, proscriptions, or descriptive stereotypes for males than females. Negative *d* scores reflect stronger prescriptions, proscriptions, or descriptive stereotypes for females than males.

Table S3

Positive Prescriptive Stereotypes, Mean Desirability Ratings, and Descriptive Stereotypes for Males and Females by Age Group in Study 3

Trait	Prescriptive *d*	Male *M*	Female *M*	Descriptive *d*	Prescriptive *d*	Male *M*	Female *M*	Descriptive *d*	Prescriptive *d*	Male *M*	Female *M*	Descriptive *d*
Males' prescriptions	Toddlers				Elementary-aged				Adolescents			
Masculine toys	1.01	7.40	4.86	0.89	1.30	7.40	4.56	1.10	--	--	--	--
Masculine appearance	--	--	--	--	1.01	6.97	4.81	0.63	0.96	6.93	4.86	0.71
Masculine interests	--	--	--	--	--	--	--	--	0.49	7.66	6.75	0.44
	Young Adults				Adults				Elderly			
Masculine toys	--	--	--	--	--	--	--	--	--	--	--	--
Masculine appearance	0.91	6.86	4.96	0.71	0.58	6.67	5.52	0.22	--	--	--	--
Masculine interests	0.75	7.83	6.41	0.49	0.51	7.7	6.7	1.11	0.48	6.77	5.73	0.92
Agentic	0.70	8.00	6.85	0.36	0.71	8.13	7.13	0.56	--	--	--	--
Intelligent	0.49	8.00	7.19	0.22	--	--	--	--	--	--	--	--
Independent	--	--	--	--	0.54	8.2	7.37	-0.10	--	--	--	--

Trait	Prescriptive *d*	Male *M*	Female *M*	Descriptive *d*	Prescriptive *d*	Male *M*	Female *M*	Descriptive *d*	Prescriptive *d*	Male *M*	Female *M*	Descriptive *d*
Females' prescriptions	Toddlers				Elementary-aged				Adolescents			
Feminine toys	-2.13	3.5	7.97	-1.55	-2.26	2.73	7.04	-1.93	--	--	--	--
Feminine appearance	-1.59	2.7	6.07	-1.48	--	--	--	--	-1.83	2.45	6.04	-1.04
Active	-0.50	7.13	8.00	-0.15	--	--	--	--	--	--	--	--
Communal	--	--	--	--	-0.72	6.47	7.56	-0.09	-0.43	6.76	7.43	-0.29
Feminine interests	--	--	--	--	-0.61	6.33	7.56	-0.55	-0.70	6.76	7.93	-0.83
Likeable	--	--	--	--	-0.48	7.57	8.11	-0.39	-0.55	7.38	8.11	-0.41
	Young Adults				Adults				Elderly			
Feminine toys	--	--	--	--	--	--	--	--	--	--	--	--
Feminine appearance	--	--	--	--	--	--	--	--	--	--	--	--
Active	--	--	--	--	--	--	--	--	--	--	--	--
Communal	-0.76	6.72	8.04	-0.28	-0.78	7.43	8.43	-0.89	-0.61	7.53	8.19	-0.37

Trait	Prescriptive *d*	Male *M*	Female *M*	Descriptive *d*	Prescriptive *d*	Male *M*	Female *M*	Descriptive *d*	Prescriptive *d*	Male *M*	Female *M*	Descriptive *d*
Feminine interests	--	--	--	--	-0.48	6.77	7.59	-0.27	--	--	--	--
Likeable)	--	--	--	--	--	--	--	--	--	--	--	--

Note. Positive *d* scores reflect stronger prescriptions or descriptive stereotypes for males than females. Negative *d* scores reflect stronger prescriptions or descriptive stereotypes for females than males.

Table S4
Negative Proscriptive Stereotypes, Mean Desirability Ratings, and Descriptive Stereotypes for Males and Females by Age Group in Study 3

Trait	Prescriptive *d*	Male *M*	Female *M*	Descriptive *d*	Prescriptive *d*	Male *M*	Female *M*	Descriptive *d*	Prescriptive *d*	Male *M*	Female *M*	Descriptive *d*
Males' proscriptions	Toddlers				Elementary-aged				Adolescents			
Feminine toys	2.13	3.5	7.97	-1.55	2.26	2.73	7.04	-1.93	0.98	2.17	4.39	-0.45
Feminine appearance	1.59	2.7	6.07	-1.48	1.79	2.20	5.74	-1.41	1.83	2.45	6.04	-1.04
Shy	0.47	3.97	5.1	0.00	--	--	--	--	0.55	3.62	4.79	-0.05
	Young Adults				Adults				Elderly			
Feminine toys	0.91	1.55	3.41	-0.08	0.81	1.67	3.17	-0.02	0.82	1.53	3.00	0.24
Feminine appearance	2.18	2.24	5.81	-1.47	2.12	2.03	5.47	-0.99	0.66	2.7	4.04	-0.19
Shy	0.71	3.03	4.56	0.18	--	--	--	--	--	--	--	--
Emotional	0.51	2.17	3.11	-0.27	--	--	--	--	--	--	--	--
Weak	0.40	2.1	2.85	-0.05	0.39	1.87	2.5	0.08	--	--	--	--

Trait	Prescriptive *d*	Male *M*	Female *M*	Descriptive *d*	Prescriptive *d*	Male *M*	Female *M*	Descriptive *d*	Prescriptive *d*	Male *M*	Female *M*	Descriptive *d*
Females' proscriptions	Toddlers				Elementary-aged				Adolescents			
Dominant	--	--	--	--	-0.45	3.17	2.26	0.38	--	--	--	--
Noisy	--	--	--	--	-0.43	5.07	3.93	0.07	-0.51	4.41	3.21	0.11
Sexually active	--	--	--	--	--	--	--	--	-0.71	3.59	1.93	0.18
Masculine toys	--	--	--	--	--	--	--	--	-0.57	4.79	3.36	0.76
	Young Adults				Adults				Elderly			
Dominant	-0.54	3.79	2.52	0.48	--	--	--	--	--	--	--	--
Noisy	--	--	--	--	-0.46	3.17	2.3	0.16	--	--	--	--
Sexually active	--	--	--	--	--	--	--	--	--	--	--	--
Masculine toys	-0.49	3.97	2.78	0.61	--	--	--	--	--	--	--	--
Rebellious	--	--	--	--	--	--	--	--	-0.52	3.2	2.08	0.60

Note. Positive *d* scores reflect stronger proscriptions or descriptive stereotypes for males than females. Negative *d* scores reflect stronger proscriptions or descriptive stereotypes for females than males.
